# Racial, ethnic, and socioeconomic disparities in clinical trial reporting for metastatic spine tumors: An exploration of North American studies

**DOI:** 10.1007/s10143-025-03343-1

**Published:** 2025-02-19

**Authors:** Ali Haider Bangash, Rose Fluss, Ananth S Eleswarapu, Mitchell S Fourman, Yaroslav Gelfand, Saikiran G Murthy, Reza Yassari, Rafael De la Garza Ramos

**Affiliations:** 1 Spine Research Group, Montefiore Medical Center, Albert Einstein College of Medicine, Bronx, NY USA; 2 Department of Neurosurgery, Montefiore Medical Center, Albert Einstein College of Medicine, Bronx, NY USA; 3 Department of Orthopedic Surgery, Montefiore Medical Center, Albert Einstein College of Medicine, 3316 Rochambeau Avenue, 3rd floor, Bronx, NY USA

**Keywords:** Metastatic spine disease, Diversity, Race, Socioeconomic status, Insurance, Employment, Vulnerability

## Abstract

**Purpose:**

The objective of this study was to evaluate the reporting of racial, ethnic, and socioeconomic data in clinical trials exploring the management of metastatic spine disease (MSD).

**Methods:**

We undertook a cross-sectional analysis of North American completed and published clinical trials registered on ClinicalTrials.gov exploring the management of patients with MSD. Data on patient demographics, trial characteristics, reporting of race and ethnicity, distribution of racial and ethnic groups, and reporting of socioeconomic measures was extracted from ClinicalTrials.gov and related publications identified through PubMed and Google Scholar searches. An exploratory data analysis was performed, followed by Pearson’s Chi-square and binary logistic regression analyses to explore associations of covariates with racioethnic reporting.

**Results:**

Out of 158 completed trials, only 8% (12 of 158) met inclusion criteria with published results. These 12 trials included a total of 1,568 patients with a mean age of 61 years. Almost half (42%; (5 of 12)) of trials did not report race, while only 17% (2 of 12) of trials reported ethnicity. In trials reporting complete racial data (*n* = 5), 77% (377 of 493) patients were White, 15% (*n* = 73) Black or African American, and 4% (*n* = 19) Asian. American Indian/Alaska Native and Native Hawaiian/Other Pacific Islander patients were severely underrepresented (0.4% and 0.2%, respectively). Of the two trials reporting ethnicity, 94% (479 of 514) patients were Not Hispanic or Latino. Sponsoring body of the trial, trial phase, intervention type, number of trial patients, or mean age of patients were not significantly associated with racioethnic reporting. Notably, no trial reported any measures of socioeconomic status.

**Conclusion:**

Our review revealed significant gaps in the reporting of racial, ethnic, and socioeconomic data in MSD clinical trials, with substantial underrepresentation of minority groups. This underrepresentation limits the generalizability of trial findings and may perpetuate health disparities. Coordinated efforts from researchers, clinicians, policymakers, and funding bodies are needed to improve diversity in future trials. Strategies such as targeted outreach, community engagement, and more inclusive eligibility criteria should be implemented to ensure that trial populations better reflect the diversity of MSD patients in the general population.

## Introduction

Spinal metastases have an estimated incidence of 16% among solid cancer tumors, and the incidence has been found to increase from 229 to 302 cases per million between 2007 and 2019 [1, 2]. Given the potential for severe pain, neurological compromise, and decreased quality of life (QOL), metastatic spine disease (MSD) requires prompt and effective management strategies [3]. Clinical trials are essential to the advancement of spinal metastases treatment modalities as the landscape of cancer care changes [4]. The generalizability and applicability of the findings of those trials to a diverse patient population, however, continues to raise concerns particularly in North America.

Previous research on patients with MSD has shown differences in outcomes based on race, ethnicity, and socioeconomic status [5, 6]. African American patients have been found to have worse outcomes including higher risk of postoperative complications, longer length of stay, and predisposition to non-routine discharges [7]. Patients having lower socioeconomic status have also been reported not to be managed by the recommended surgical intervention [8] whereas private insurance status lead to increased odds of surgical intervention [9]. Moreover, length of stay and postoperative complications have been found to be significantly reduced for patients dwelling in ZIP codes with higher levels of education [10]. The findings highlight how crucial it is to have a varied representation of racial, ethnic, and socioeconomic groups of patients in clinical trials in order to guarantee that new treatments are studied and distributed fairly across all patient groups.

The objective of this study was to evaluate the reporting of racial, ethnic, and socioeconomic data in clinical trials exploring the management of spinal metastases conducted in North America. We sought to evaluate the existing status of diversity reporting in spinal metastasis research to pinpoint aspects that require attention and tender recommendations to strengthen future studies around this subject to be more inclusive.

## Materials and methods

### Study design and data set

We conducted a cross-sectional analysis of North American clinical trials exploring management of patients with metastases to the spine registered on the ClinicalTrials.gov database. The data collection was performed on July 29th, 2024. ClinicalTrials.gov is a publicly accessible online database of clinical research studies conducted worldwide [11]. 

We identified relevant clinical trials using the keywords “spine tumor OR spinal tumor OR spine cancer OR spinal cancer OR metastatic spine disease OR metastatic spinal disease OR metastatic spine tumor OR metastatic spinal tumor”. Institutional Review Board supervision was not required for this study as we extracted de-identified data from published trials.

### Research objective

The primary objective of our study was to evaluate the reporting of racial, ethnic, and socioeconomic data in clinical trials that were undertaken to explore the management of spinal metastasis.

### Collected data

For the selected clinical trials, we collected the following data: Number of patients, age, primary histology type, location of spinal metastases, type of intervention (Surgery, radiation, systemic), reporting of race and ethnicity, distribution of racial groups (White/Caucasion, Black/African American, Asian, Native Hawaiian or Other Pacific Islander, American Indian or Alaska Native, More than 1 race, Unknown or not reported), distribution of ethnic groups (Hispanic or Latino, Not Hispanic or Latino, Unknown or Not Reported) and reporting of socioeconomic measures (Insurance status, income, employment status, occupation, primary language, Social Vulnerability Index, Area Deprivation Index).

### Data collection approach

We obtained participant enrollment data through two approaches. For trials with results reported directly on ClinicalTrials.gov, we extracted the data from the database. For trials without reported results on ClinicalTrials.gov, we conducted a search of PubMed and Google Scholar databases to identify related publications. The search parameters included the trial’s title, principal investigator, and associated institute. We used terms such as “Race,” “Ethnicity,” “African American,” “Black,” “Caucasian,” and “White” to filter documents.

### Inclusion criteria

Clinical trials were included if they met the following criteria: Recruited patients had metastatic tumors to the spine, studies were were undertaken in the North American region and were completed at the time of data collection with their data published.

### Exclusion criteria

Clinical trials were excluded if they included patients with primary central nervous system tumors or had their results not published at the time of our data collection search.

### Statistical analysis

We performed an exploratory data analysis. Categorical variables, including the reporting of race and ethnicity and the distribution of different racial and ethnic groups, were expressed as percentages of the total. Continuous variables, such as age, were expressed as mean (with standard deviation, where applicable) or median (with range, where applicable). We developed charts to visualize the distribution of racial and ethnic groups in the reported trials. Pearson’s Chi-square analysis was undertaken to appreciate the association of categorical variables with race and ethnicity reporting. Binary logistic regression analysis was undertaken to elucidate the association of continuous variables with race and ethnicity reporting. The analysis was undertaken using IBM SPSS (version 26).

## Results

Our initial search yielded 593 clinical trials registered worldwide, out of which 53% (317 of 593) clinical trials were registered in North America (USA = 265 and Canada = 52]). Out of those North American trials, only 50% (*n* = 158) were reported to be completed. Of these, the results of only 8% (*n* = 12) trials were published and included in our final analysis. (Table 1)

**Table 1:** North American trials exploring management of spinal metastasis with published results

Study and year	Registration Number	Sponsoring Body	Patients	Age	Race and Ethnicity of Patients	Intervention	Outcome
Patsalides A et al., 2016	NCT01637766	Academic university hospital	*n* < 10	Median 48 years, range 19–76	Not reported	Spinal intraarterial chemotherapy	No catheterization-related, angiography-related, or local toxicity adverse events observed One Grade 4 adverse event (neutropenic fevers) and one Grade 2 adverse event (nausea) reported Local tumor control achieved in 8 of 9 patients 7 of 8 patients with local tumor control showed disease progression at other spinal sites Tumor progression at the treated level observed in 1 patient (leiomyosarcoma case), leading to discontinuation of the treatment
Albert Einstein College of Medicine, 2024	NCT02527304	Academic university hospital	24	Mean 66.5 years, range 32–92	Race: 16.7% White (*n* < 10), 50% Black or African American (*n* = 12), 20.8% More than 1 race (*n* < 10), 12.5% Unknown or Not Reported (*n* < 10) Ethnicity: Not reported	Adaptive staged SBRT	No grade 3 or higher treatment-related toxicity 100% 2-year OS 92.3% 2-year PFS
University of Texas Southwestern Medical Center, 2022	NCT00855803	Academic university hospital	35 (29 patients completed study protocol.)	Median 60 years, range 40–77	Race: 77.1% White (*n* = 27), 20% Black or African American (*n* < 10), 2.9% Asian (*n* < 10) Ethnicity: Not reported	SBRT + vertebroplasty	5-year survival = Median of 9 months (0–63.4 months) 57.1% patients with complete pain response at 3 months
Redmond KJ et al., 2020	NCT01752036	Academic university hospital	35	Median 63 years, range 21–75	Race: 68.6% White (*n* = 24), 22.9% Black or African American (*n* < 10), 8.6% Asian (*n* < 10) Ethnicity: Not reported	SBRT	Local control rate = 90.0% (95% CI, 76-98%) Median OS = 14.3 months (95% CI: 7.6–43)
Memorial Sloan Kettering Cancer Center, 2019	NCT02320825	Cooperative group	16	Mean 57.44 years, range 45–72	Race: 87.5% White (*n* = 14), 6.3% Black or African American (*n* < 10), 6.3% Unknown or Not Reported (*n* < 10) Ethnicity: Not reported	SRS	All-cause mortality rate of 87.5% in hypofractionated arm vs. 62.5% in single-fraction arm
Schoenfeld AJ et al., 2022	NCT03224650	Cooperative group	202	Mean 60.5 years; Standard deviation of 12	Race [Incomplete reporting]^¶^: 86% White (*n* = 174 Ethnicity: Not reported	Validation of NESMS	NESMS outperformed all spinal metastases treatment outcomes prognostication scoring scales
University of Florida, 2012	NCT00631670	Academic university hospital	21	Mean 58.5 years; Standard deviation of 15.9	Race: Not reported Ethnicity: Not reported	SBRT	OS rate at 1 year = 23.8% Local control rate of tumor sites at 1 year = 96%
Rades D et al., 2016	NCT02189473	Cooperative group	203	Median 68 years	Race: Not reported Ethnicity: Not reported	3D-conformal RT (comparison of 2 regimens)	Similar overall response rates at 1 month (87.2% vs. 89.6%, *p* = 0.73) Comparable ambulatory rates at 1 month (71.8% vs. 74.0%, *p* = 0.86) No significant difference in PFS and OS at 3 and 6 months Well-tolerated toxicity profiles for both regimens
Radiation Therapy Oncology Group, 2021	NCT00922974	Cooperative group	383	Median 63 years, range 23–93	Race: 80.4% White (*n* = 308), 11.7% Black or African American (*n* = 45), 3.9% Asian (*n* = 15), 0.3% Native Hawaiian or Other Pacific Islander (*n* < 10), 0.5% American Indian or Alaska Native (*n* < 10), 0.5% More than 1 race (*n* < 10), 2.6% Unknown or Not Reported (*n* = 10) Ethnicity: 91.9% Not Hispanic or Latino (*n* = 352), 5.2% Hispanic or Latino (*n* = 20), 2.9% Unknown or Not Reported (*n* = 11)	IGR / SRBT vs. EBRT	Pain relapse rate through 2 years = 91.7% (84.9 to 98.6) in IGR / SRBT group vs. 96.9% (92.6 to 100) in EBRT group Similar rates of VCF and spinal cord damage through 2 years
Barzilai O et al., 2024	NCT01825161	Cooperative group	280	Mean 57.9 years, range 18–75	Race: Not reported Ethnicity: Not reported	Exploration of postsurgical QoL of EPOSO trial patients	Significant HRQOL improvements at all time points up to 2 years post-treatment Physical component score improved by 6.4 points at 2 years (*p* < 0.001) Mental component score improved by 4.6 points at 2 years (*p* = 0.004) EQ-5D score improved by 0.24 points at 2 years (*p* < 0.001) Pain score decreased by 2.6 points, stable from 6 weeks to 2 years (*p* < 0.001) Overall QoL score improved by 16.8 points at 2 years (*p* < 0.001)
Berenson J et al., 2021	NCT00211237	Private medical company	131	Mean 63.9 years	Race [Incomplete reporting]^¶^: 87.02% White (*n* = 114), 6.87% Black or African American (*n* < 10), 1.52% Asian (*n* < 10), 2.29% Other (*n* < 10) Ethnicity: 96.94% Not Hispanic or Latino (*n* = 127)^§^, 0.76% Hispanic or Latino (*n* < 10), 2.29% Other (*n* < 10)	BK vs. conservative management	At 1 month, kyphoplasty group had 8.3 point RDQ improvement (*p* < 0.0001) vs. 0.1 point in non-surgical group (*p* = 0.83) [Treatment effect − 8.4 (*p* < 0.0001)] Kyphoplasty group had 8.4 point SF-36 improvement (*p* < 0.0001) and 11.1 point mental component improvement (*p* < 0.0001) vs. non-surgical group. Kyphoplasty group had 15.3 point KPS improvement (*p* < 0.0001) and 6.3 fewer limited activity days (*p* < 0.0001) vs. non-surgical group. At 7 days, kyphoplasty group had 3.5 NRS pain score vs. 7.0 in non-surgical group (*p* < 0.0001) [Treatment effect − 3.5 (*p* < 0.0001)]. Adverse events similar between groups at 1 month
Sahgal A et al., 2021	NCT02512965	Cooperative group	229	Mean 63.86 years; Standard deviation of 12.38	Race: Not reported Ethnicity: Not reported	SBRT vs. EBRT	Superior response of pain by SBRT (RR = 1·33, 95% CI” 1·14–1·55; *p* = 0·0002) No difference in OS at 3 and 6 months

¶ The respective percentages as shared in the published trial data do not equal 100%

§ The number of non-hispanic or non-latino patients in the Berenson J et al., 2021 trial is extracted indirectly by applying arithmetic operation on the published available ethnicity data in that trial

SBRT = Stereotactic Body Radiation Therapy, OS = Overall Survival, PFS = Progression-free survival, SRS = Stereotactic Radiosurgery, NESMS = New England Spinal Metastasis Score, RT = Radiotherapy, IGR = Image-Guided Radiosurgery, EBRT = External Beam Radiation Therapy, VCF = Vertebeal Compression Fracture, QoL = Quality of Life, EPOSO = Epidemiology, Process, and Outcomes of Spine Oncology, HRQOL = Health-related Quality of Life, EQ-5D = EuroQol-5 Dimension, RR = Risk ratio, BK = Balloon Kyphoplasty, RDQ = Roland-Morris disability questionnaire, SF-36 = 36-item short form health survey (SF-36) score, KPS = Karnofsky performance status, NRS = Numeric Rating Score

### General patient demographics and trial characteristics

The trials were conducted between 2005 and 2022 with participating site locations for these trials scattered throughout North America including New York, Boston, Dallas, Baltimore and Stanford as well as Vancouver, Toronto and Montreal. Moreover, 42% (5 of 12) trials were also simultaneously undertaken in healthcare centers across Europe, Australia, and the Middle East, in addition to the respective North American sites [12–16]. Only 8% (1 of 12) trials were sponsored by a private medical company [15]. (Table 1)

A total of 1568 patients were reported on with a mean age of 61 years and 46% (727 of 1568) patients being female. Only 17% (2 of 12) of trials were observational (phase IV) [14, 17] whereas phase II design was the commonest interventional approach amongst the included trials, adopted by 33% (*n* = 4) trials [18–21]. Two-thirds (67%; *n* = 8) of trials explored radiotherapy [12, 13, 16, 18–22]. (Table 2)

**Table 2:** Phase and intervention type of north American trials exploring management of spinal metastasis

Phase status		
	**Phase 1**	1 (8.33%)
	**Phase 1/Phase 2**	ZERO
	**Phase 2**	4 (33.33%)
	**Phase 2/Phase 3**	2 (16.67%)
	**Phase 3**	3 (25%)
	**Phase 4**	2 (16.67%)
**Intervention type** ^**a**^		
	**Radiation**	8 (66.67%)
	**Surgery**	1 (8.33%)
	**Drug**	1 (8.33%)

^a^10 of 12 included trials were of interventional type

One quarter (25%; 3 of 12) trials neither reported the primary histology type nor the location of spinal metastases [18, 21, 22] whereas 42% (*n* = 5) of trials did not report the location of spinal metastases [12, 15, 18, 21, 22]. Lung was found to be the commonest primary cancer site^12.16, 17, 19, 20^ and the thoracic spine was inferred to be the most common region for the tumors to metastasize [14, 16, 17, 20, 23]. 

### Race and ethnicity

**Fig. 1:**
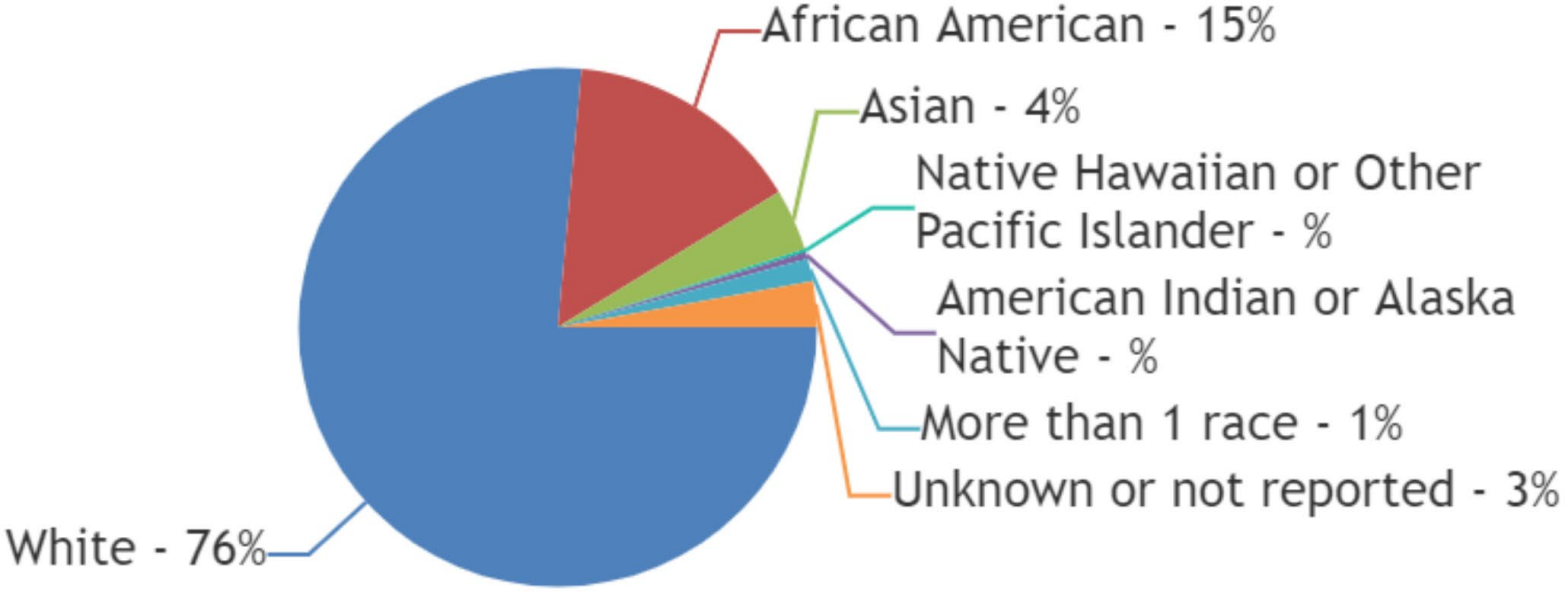
Racial diversity of patients included in trials exploring management of spinal metastasis

58% (7 of 12) trials reported the race of the patients [13, 15, 17–20, 22] and only 17% (2 of 12) trials reported the ethnicity of the patients.^13,15^ 16.67% (2 of 12) trials reported incomplete racial data [15, 17]. Out of a total of 493 patients reported in 5 trials that published complete racial data [13, 18–20, 22], 76% (377 of 493) were reported to be White, 15% (*n* = 73) were Black or African American and 4% (*n* = 19) were Asian. Only 0.2% (*n* = 1) were Native Hawaiian or Other Pacific Islander whereas only 0.4% (*n* = 2) were American Indian or Alaska Native. One-percent of patients ( *n* = 7) were reported to have more than one race whereas the race of 3% (*n* = 14) of patients was reported to be unknown or not reported. [Figure 1]

Sponsoring body of the trial (χ2 = 0.891; p-value = 0.64), trial phase (χ2 = 2.057; p-value = 0.725), or intervention type (χ2 = 1.029; p-value = 0.598) were not significantly associated with race reporting. Moreover, a unit increase in either the number of trial patients [B= -0.004 (S.E: 0.005); Exp(B) = 0.996; p-value = 0.43) or mean age of patients [B = 0.156 (S.E: 0.143); Exp(B) = 1.169; p-value = 0.275) was not significantly associated with an increase in the odds of race reporting.

**Fig. 2:**
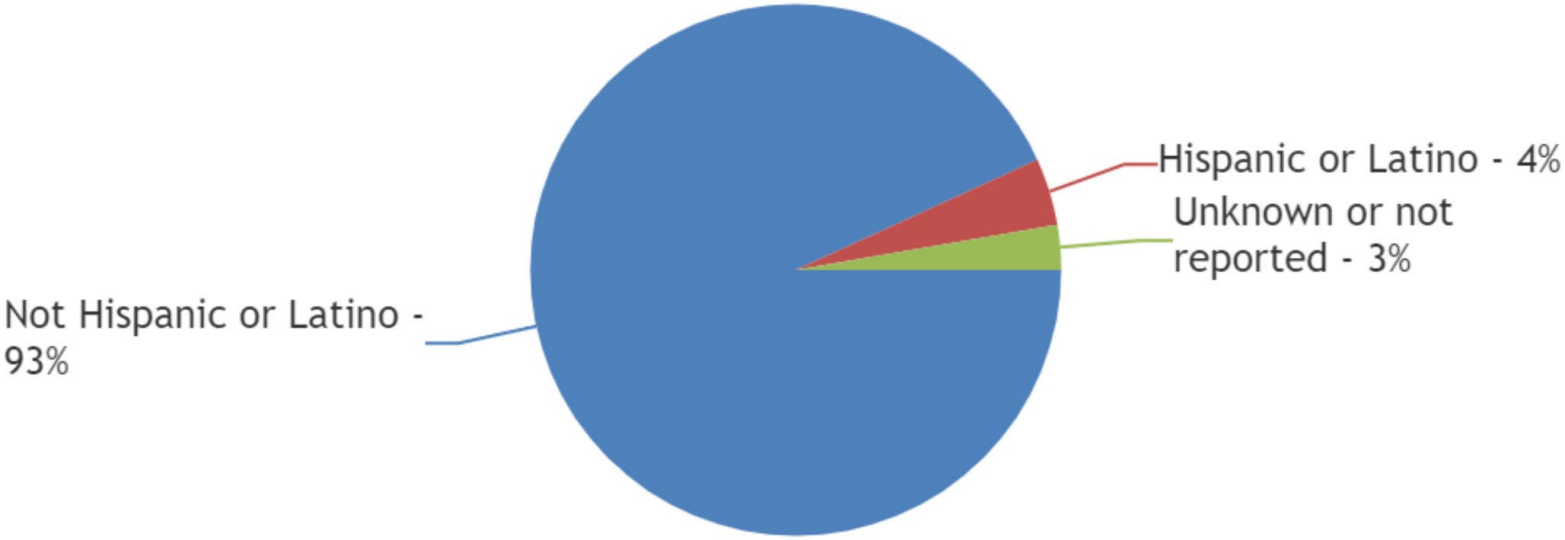
Ethnic diversity of patients included in trials exploring management of spinal metastasis

Out of a total of 514 patients reported in 2 trials that published ethnic data [13, 15], 93% (*n* = 479) were Not Hispanic or Latino, 4% (*n* = 21) were Hispanic or Latino, and the ethnicity of 3% (*n* = 14) patients was reported to be Unknown or Not Reported. [Figure 2]

Sponsoring body of the trial (χ2 = 6; p-value = 0.05), trial phase (χ2 = 3.6; p-value = 0.463), or intervention type (χ2 = 0.9; p-value = 0.638) were not significantly associated with ethnicity reporting. Moreover, a unit increase in either the number of trial patients [B = 0.011 (S.E: 0.009); Exp(B) = 1.011; p-value = 0.206) or mean age of patients [B = 0.176 (S.E: 0.277); Exp(B) = 1.193; p-value = 0.525) was not significantly associated with an increase in the odds of race reporting.

### Measures of socioeconomic status

Insurance status, income, employment status, occupation, primary language, Social Vulnerability Index, Area Deprivation Index, or other measures of socioeconomic status were not reported by any of the included trials.

## Discussion

Significant gaps in the reporting of racial, ethnic, and socioeconomic data in clinical trials examining the management of MSD were found by our review. The results showed a worrying pattern of minority group underrepresentation and a dearth of socioeconomic data, which might restrict the applicability of trial findings as well as perpetuate existing health disparities [24]. 

Of the 12 trials included in our analysis, just over half (58%) reported the race of patients, and only 17% reported ethnicity. This scarcity of reporting that was uncovered in MSD patient population trials aligns with the findings of earlier research explorations that investigated the state of racial, ethnic and socioeconomic reporting in other oncological domains. For example, Taha et al. discovered that data on race or ethnicity were only disclosed in 28% of brain tumor clinical trials [25]. Racial and ethnic data was not reported in as many as 53% of precision-oncology studies for patients with breast, colorectal, lung, and prostatic cancer [26]. The ability of healthcare professionals involved in managing MSD to critically evaluate whether the trial populations adequately reflect the demographics of patients with spinal metastases in the general community is hampered by the lack of this essential data [27]. 

White patients constituted a considerable overrepresentation in the studies that provided racial data, accounting for 76% of the research population. This overrepresentation is consistent with findings from earlier oncological research across all neurological cancers, including those published by Reihl SJ et al. where they found minority racial groups to be severely under-represented and reported a mortality specific over-accrual for White Caucasians in neuro-oncology trials [28]. The small proportion of patients from minority groups, including Black or African American patients (15%) and Asian patients (4%), raises concerns about the applicability of trial results to these populations [29]. 

Particularly concerning is the almost complete absence of American Indian or Alaska Native patients (0.4%) and Native Hawaiian or Other Pacific Islander patients (0.2%) in trials involving patients with MSD. Due to this extreme underrepresentation, there may be little information about how spinal metastasis therapies impact these communities, which might exacerbate already-existing health inequities [30]. Historically, American Indian and Alaska Native patients have been reported to experience significant barriers to cancer screening and treatment, leading to increased vulnerability to cancer-related morbidity and mortality [31]. These long-standing healthcare disparities that limit their access to necessary care have lead to sub-optimal cancer screening and poorer cancer outcomes [32–34]. Therefore, efforts should be undertaken to assimilate especially these marginalized communities into the mainstream research avenues [35]. 

Notably, none of the included trials reported any measures of socioeconomic status, such as insurance status, income, or employment. Similarly, the primary language was not reported. Social indices were not reported by any trial, either. Given that other research has shown the influence of socioeconomic variables on cancer outcomes, these measures not being available is noteworthy. Private insurance has been reported to be associated with decreased odds of non-routine discharge and length of stay [36]. Associated financial strain has been reported to be associated with anxiety and lower QOL amongst patients with MSD [37, 38]. Insufficient socioeconomic data reporting in spinal metastasis trials shall make it harder to identify disparities among outcomes and care access, which might impede the development and implementation of focused efforts aimed at enhancing health equality across a range of patient demographics [39]. 

These underrepresentation and reporting discrepancies are probably caused by a variety of factors. Language hurdles, financial restrictions, mistrust of the medical system, and lack of information are some of the challenges that lower socioeconomic and minority groups face while trying to get involved in clinical trials [40]. Furthermore, by focusing recruiting efforts in areas with less varied populations or by imposing too rigorous eligibility requirements, trial designs may unintentionally exclude certain population groups [41, 42]. 

It is imperative to note the regulatory context surrounding clinical trial reporting and diversity. The Food and Drug Administration Amendments Act (FDAAA) of 2007, improved in 2017, mandated the reporting of clinical trial results [43]. This legislation aimed to increase transparency and accessibility of clinical trial data. More recently, in April 2022, the FDA issued new draft guidance on diversity plans to improve enrollment of participants from underrepresented racial and ethnic populations in clinical trials [44]. This guidance recommended that sponsors of medical products develop and submit a Race and Ethnicity Diversity Plan to the FDA early in clinical development, which shall be implemented by early 2025 [45]. These regulatory efforts underscore the growing recognition of the importance of both comprehensive reporting and diverse representation in clinical trials. However, as our systematic review demonstrated, there remains a significant gap between these regulatory ideals and the reality of reporting practices in trials for metastatic spine disease. It is also important to note that while racial and ethnic diversity in clinical trials has gained increasing attention, the reporting of socioeconomic status remains rare, typically limited to retrospective studies that specifically focus on it [10, 46]. These shortcomings highlight the need for more rigorous implementation and enforcement of comprehensive guidelines.

### Strategies to improve inclusivity in metastatic spine tumors trials

To address the aforementioned deficiencies, several strategies can be implemented. It would be advantageous for funding organizations and journal editors to mandate the submission of thorough demographic and socioeconomic data in order to approve grants or publish articles [47]. Researchers ought to work with healthcare practitioners that serve a variety of groups and conduct focused outreach to underprivileged areas [48, 49]. Building trust and increasing participation from underrepresented groups can be achieved by involving patient advocates and community leaders in trial design and recruitment [50, 51]. Moreover, diversifying the clinical trial workforce and investigators may facilitate the design of more inclusive trials and enhance interactions with a range of patient populations [52]. Patients who do not speak English well can participate more easily if research materials are available in many languages and interpreters are accessible [53, 54]. Reduction of financial obstacles to trial participation can be achieved by providing appropriate remuneration for participation and reimbursement for travel expenses [55, 56]. Furthermore, enhancing diversity may be achieved by putting in place more inclusive eligibility criteria and taking into account practical trial designs that mirror real-world patient groups [57]. It is also possible to increase recruitment from underprivileged groups by raising knowledge of clinical trials among patients and healthcare professionals in these locations [58, 59]. 

Opportunities and challenges arise from the geographic dispersion of included trials, which included many locations throughout North America as well as Europe, Australia, and the Middle East. Although this wide reach may have contributed to more diversity, our results indicated that the root cause of underrepresentation has not been sufficiently addressed. This emphasizes that, regardless of the trial’s geographic reach, deliberate, focused efforts are required to increase diversity [60]. The predominance of radiotherapy-focused trials (67%) in our sample reflects the importance of this modality in managing spinal metastases [61]. However, to promote fair advancement in all facets of MSD management, it is imperative that equitable representation of all racial and socioeconomic patient groups is ensured in all trials exploring the entire spectrum of treatment modalities, including surgical and systemic treatments, for MSD [62]. 

Our systematic review has several strengths, including its comprehensive search strategy and its focus on the critical issue of diversity reporting. By highlighting the gaps in racial, ethnic, and socioeconomic reporting, we provided valuable insights for future research directions. However, our review also had limitations that warrant consideration. The small number of included trials (*n* = 12) with published results limited the generalizability of our findings. Our focus on North American trials, while allowing for a targeted analysis, may not have reflected global trends in diversity reporting for spinal metastasis research. Additionally, the retrospective nature of our systematic review made it challenging to fully understand the reasons behind the observed gaps in diversity reporting. Potential biases in our analysis included selection bias, as we only included trials with published results, which may not have represented all completed trials. There may also have been reporting bias in the original trials, where certain demographic information might have been collected but not reported. Furthermore, a temporal bias could also have been into play, as reporting practices might have changed over the time period covered by the included trials. Lastly, there could have been a geographic bias within North America, as trials conducted in more diverse urban centers might have different demographic compositions compared to those in less diverse areas.

Notwithstanding, our research offered insightful information on the status of diversity reporting in MSD clinical trials. The results are consistent with more general patterns in cancer research, pointing to a systemic vulnerability that transcends this particular patient population [63]. In order to ensure that MSD trial findings may be adopted broadly and to advance health equality in cancer care, it is imperative that these inequities be addressed [64]. 

## Conclusion

Our study revealed significant room for improvement in the reporting of racial, ethnic, and socioeconomic data in MSD trials as our understanding of how novel therapies may influence various patient populations is limited by the underrepresentation of minority groups and the absence of socioeconomic data. A coordinated effort by researchers, physicians, legislators, and funding organizations will be required to bridge these gaps. We may endeavor to discover more fair and effective therapies for all patients with spinal metastases, regardless of their socioeconomic status or race, by improving the inclusivity in future trials. Future studies should concentrate on putting diversity improvement measures into practice and assessing their effectiveness, as well as investigating the effects of these initiatives on patient outcomes and health inequities in the management of MSD.

## Data Availability

No datasets were generated or analysed during the current study.
